# Mechanisms of action of retinal microglia in diabetic retinopathy (Review)

**DOI:** 10.3892/ijmm.2025.5643

**Published:** 2025-09-22

**Authors:** Yuyang Bai, Xinrong Wang, Fan Qi, Xiaoyang Zuo, Gang Zou

**Affiliations:** 1 Third Clinical Medical College, Ningxia Medical University, Yinchuan, Ningxia Hui Autonomous Region 750001, P.R. China; 2 Department of Ophthalmology, Ningxia Eye Hospital, People's Hospital of Ningxia Hui Autonomous Region, Third Clinical School of Medicine, Ningxia Medical University, Yinchuan, Ningxia Hui Autonomous Region 750001, P.R. China

**Keywords:** diabetic retinopathy, microglial cell, blood-retinal barrier, neurovascular unit, targeted therapy

## Abstract

Diabetic retinopathy (DR), a leading cause of blindness in diabetic microvascular complications, is pathologically associated with the dynamic regulation of retinal microglia. The present review systematically elucidated the dual roles of microglia in DR pathogenesis. Under physiological conditions, microglia maintain blood-retinal barrier (BRB) integrity by phagocytosing metabolic debris and secreting neurotrophic factors. However, hyperglycaemic stress induces pathological M1 polarization, triggering a cytokine storm (TNF-α and IL-1β) via the Toll-like receptor 4/myeloid differentiation primary response 88/NF-κB signalling axis, which synergizes with proangiogenic factors (such as VEGF and insulin-like growth factor 1) to exacerbate BRB breakdown and pathological neovascularization. Notably, activated microglia amplify inflammatory cascades through astrocyte-Müller cell interaction networks, accelerating neurovascular unit dysfunction. Emerging therapeutic strategies targeting microglial polarization homeostasis (such as promoting M2 anti-inflammatory phenotypic shifts) and blocking critical inflammatory signalling pathways present novel opportunities for developing multitarget therapeutic agents with combined neuroprotective and anti-vasopermeability properties. By elucidating microglial heterogeneity and intercellular regulatory networks, the present review highlighted the importance of precise modulation of immune homeostasis in DR management, providing a theoretical foundation for overcoming the limitations of single-target therapies.

## 1. Introduction

**Figure 1:**
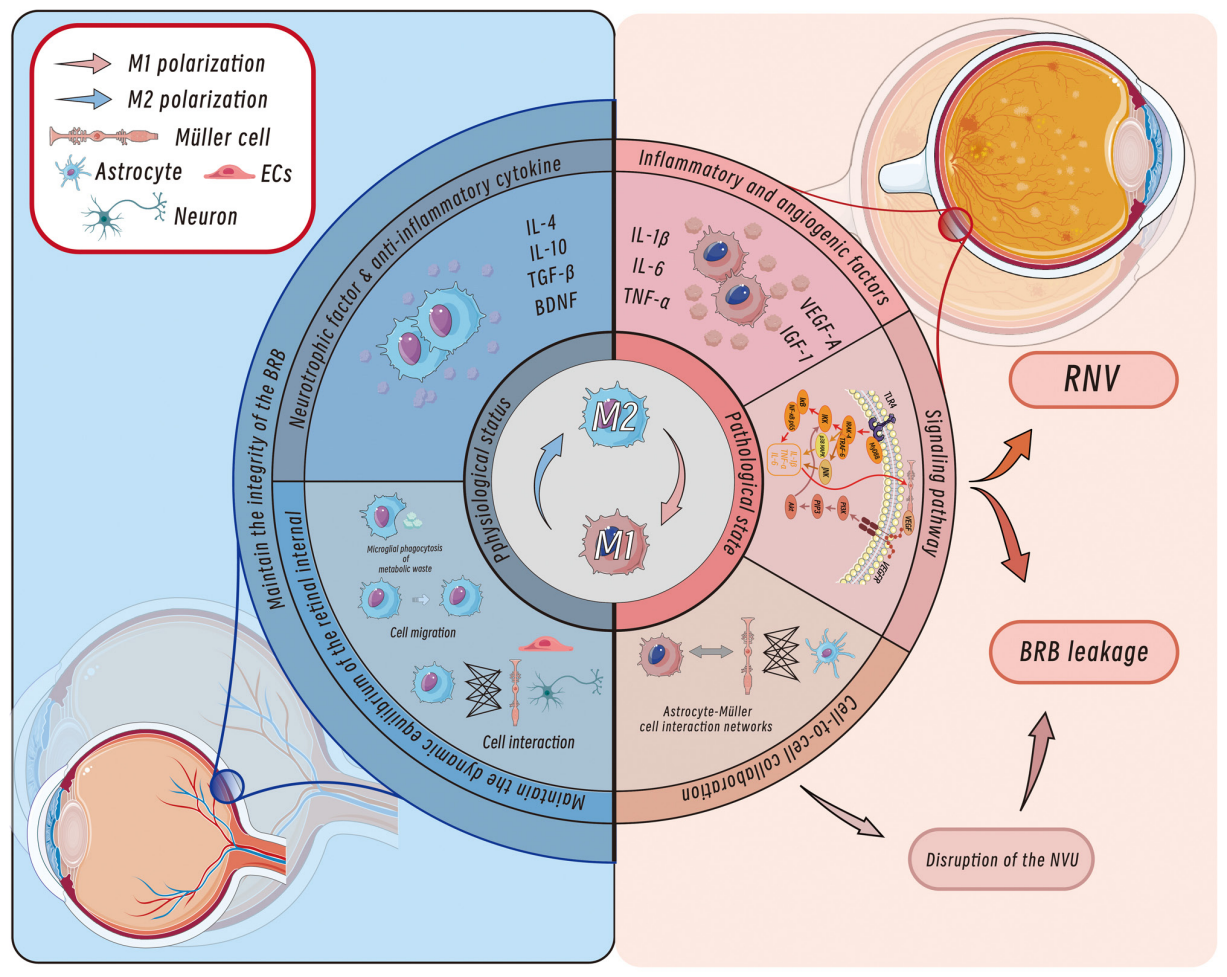
Mechanisms of vascular and neuronal modulation of retinal microglia in DR. Under physiological conditions, M2 polarization dominates, releasing anti-inflammatory and neurotrophic factors such as IL-4, IL-10, TGF-β and BDNF. Microglia maintain retinal homeostasis via phagocytosis of metabolic waste, cell migration and interactions with Müller and astrocytes, preserving the BRB. In pathological states, M1 polarization prevails, producing pro-inflammatory and angiogenic factors, including IL-1β, IL-6, TNF-α, VEGF-A and IGF-1. These factors disrupt the BRB, causing BRB leakage and RNV. The imbalance between M1 and M2 polarization also disrupts cellular interactions, retinal homeostasis and the neurovascular-immune network, ultimately damaging the NVU. DR, diabetic retinopathy; BDNF, brain-derived neurotrophic factor; BRB, blood-retinal barrier; RNV, retinal neovascularization; IGF-1, insulin-like growth factor 1; NVU, neurovascular unit.

The prevalence of diabetes mellitus (DM), a global chronic metabolic disorder, is rapidly increasing in developing countries, with >400 million current global cases; approximately one-third of individuals with DM develop diabetic retinopathy (DR) (1-4). DR is characterized by progressive neurovascular damage in the retina and is clinically classified into non-proliferative DR (NPDR) and proliferative DR (PDR); the latter leads to irreversible vision loss through complications such as pathological neovascularization and vitreous haemorrhage, with global PDR cases projected to surpass 100 million by 2045 (5-9). Previous studies have reported the dual role of retinal microglia, the resident immune sentinels of the central nervous system (CNS), in DR pathogenesis (2,10). While maintaining homeostasis through metabolic waste clearance and neuroprotection, the hyperglycaemia-induced aberrant activation of microglia exacerbates inflammatory cascades and vascular injury (Fig. 1) (11-14). The application of single-cell sequencing technologies has further elucidated microglial heterogeneity and their interactive networks with retinal endothelial cells and astrocytes, providing novel insights into the molecular mechanisms underlying DR (14).

**Figure 2:**
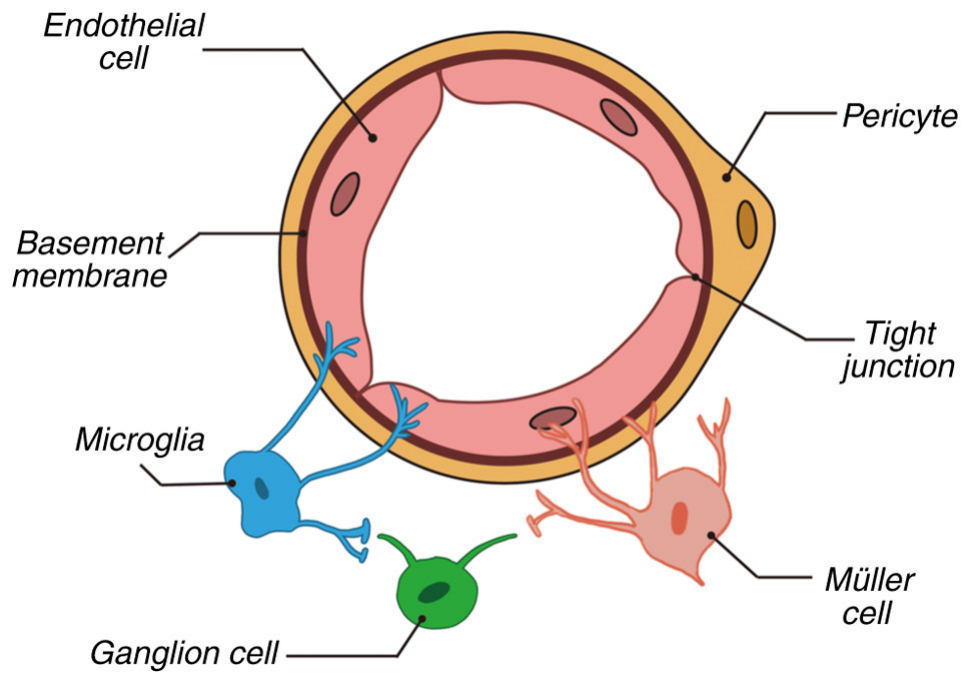
Composition of neurovascular unit. The neurovascular unit comprises endothelial cells, pericytes, basement membrane, tight junctions, microglia, Müller cells and ganglion cells, which together maintain retinal homeostasis. Endothelial cells line blood vessels and form tight junctions regulating substance exchange. Pericytes embedded in the basement membrane support vascular stability. Microglia serve as resident immune cells, while Müller cells provide metabolic and structural support to neurons. Ganglion cells transmit visual information. Collectively, these components regulate the blood-retinal barrier, blood flow and support neuronal function.

Microglia, the resident immune cells of the CNS, originate from erythromyeloid progenitors in the yolk sac during embryogenesis. These cells possess unique self-renewal capabilities and maintain homeostatic density through localized proliferation throughout their lifespan (13). In 1919, Pio del Rio-Hortega first defined microglia as a distinct population separate from neurons and other glial cells, confirming their presence in the retina (13). As members of the mononuclear phagocyte system, microglia continuously perform immune surveillance in adulthood, stabilizing the retinal microenvironment by clearing cellular debris, pathogens and abnormal proteins (15,16). A previous study reported that the functional states of microglia are closely linked to metabolic regulation and interactions with the neurovascular unit (NVU), resulting in significant dynamic alterations in pathological contexts such as DR (17). The NVU is a multicellular network of endothelial cells, pericytes, glia, neurons and the extracellular matrix that regulates blood flow and molecular exchange (Fig. 2) (18).

As a critical component of the mononuclear phagocyte system, retinal microglia colonize the retina during embryogenesis and participate in the regulation of vascular development (15,19,20). Postnatally, microglia undergo dynamic migration to establish stable spatial distribution patterns. In adulthood, microglia are widely distributed across inner retinal layers (for example, the ganglion cell layer) and outer layers (such as the outer nuclear layer), with notably reduced density in perivascular regions (21-24). This distribution is closely associated with neuronal functional activity and metabolic demands, as microglia in areas with high neuronal density predominantly exhibit a ramified morphology and maintain tissue homeostasis through continuous microenvironmental surveillance (16,25).

Under physiological conditions, retinal microglia sustain a dynamic equilibrium by forming a functional interaction network with neurons, vascular endothelial cells and Müller cells. The roles of retinal microglia include phagocytosing pathogens, clearing cellular debris and modulating synaptic plasticity (16,24,26). Notably, the distribution and activation states of retinal microglia are highly context dependent. For example, in pathological contexts such as DR, microglia display marked abnormalities in quantity and spatial positioning, characterized by perivascular infiltrative clustering and proinflammatory phenotype activation. These alterations suggest that disruption of microglial homeostasis may drive disease progression through mechanisms such as vascular permeability regulation and neuroinflammatory cascades (17). Given that microglial interactions with endothelial cells are integral to vascular integrity, their dysregulation may directly compromise the structural and functional stability of the blood-retinal barrier (BRB) (27).

The BRB, comprising the inner (iBRB) and outer barriers, safeguards retinal homeostasis through its NVU (18,28-30). In DR, hyperglycaemia-driven oxidative stress disrupts the iBRB, leading to pathological neovascularization characterized by fragile, leaky vessels that exacerbate macular oedema, haemorrhage and tractional retinal detachment (31,32). Central to this process are retinal microglia, the resident retinal immune sentinels, which serve dual roles in DR progression. Under physiological conditions, microglia maintain BRB integrity by modulating ocular immune privilege, phagocytosing debris and stabilizing the vascular tone through dynamic NVU interactions (33-35). However, in DR, chronic hyperglycaemia activates microglia in a proinflammatory state, triggering innate immune responses that amplify neurovascular injury, disrupt BRB function and recruit systemic immune mediators (36-39). Paradoxically, microglia also retain protective plasticity, migrating to subretinal spaces to clear cytotoxic debris, supporting retinal pigment epithelium (RPE) integrity and mitigating vascular leakage (40,41). This duality positions microglia as key regulators of retinal immune-metabolic crosstalk, balancing destructive inflammation with reparative functions and making them pivotal therapeutic targets in DR pathogenesis.

## 2. Phenotypic polarization of microglia in DR

Microglia can be classified into multiple subtypes based on morphological features, gene expression profiles, anatomical localization and functional states. The classical categorization distinguishes between M1-like and M2-like phenotypes (42). M1-polarized microglia, characterized by the expression of markers such as inducible nitric oxide synthase, CD16 and CD32, represent a 'classically activated' proinflammatory state. M1-polarized microglia secrete high levels of IL-1β, IL-6 and TNF-α. By contrast, M2-polarized microglia express markers such as CD206, arginase-1 and chitinase-like 3, exhibiting an 'alternatively activated' anti-inflammatory and tissue-reparative phenotype through the release of IL-4, IL-10 and TGF-β (43-45). In DR, microglial activation becomes imbalanced, with hyperactivated M1-like microglia predominating and driving retinal inflammation and injury (17). Consequently, modulating microglial activation may represent a key therapeutic strategy for DR. Using streptozotocin (STZ)-induced type 1 diabetes models and spontaneous type 2 diabetes models, studies have found that drugs such as minocycline and genistein can effectively inhibit microglial activation, thereby alleviating the symptoms of diabetic retinopathy (46-48). Additionally, certain pharmacological agents, such as minocycline and pioglitazone, have been shown to have retinal protective effects by regulating microglial activation states (49). These findings underscore the importance of investigating microglial activation mechanisms for developing novel DR therapies.

Advances in single-cell RNA sequencing have improved the understanding of microglial transcriptional and metabolic dynamics (50). For example, homeostatic microglia exhibit a unique transcriptional signature involving genes such as the purinergic receptor P2Y12 (P2RY12) and C-X3-C motif chemokine receptor 1 (51,52). In addition to these known factors, previous studies have identified the spalt-like transcription factor 1 as a key regulator essential for maintaining microglial identity and function, potentially restricting the transition of microglia into the disease-associated microglia (DAM) state by repressing the expression of certain DAM marker genes, such as Spp1/Osteopontin (53,54).

Although individual microglia have a finite lifespan, their self-renewal capacity compensates for cellular attrition, preserving region-specific population densities (13). Given their morphological plasticity and subtype diversity, identifying highly specific markers is crucial for precise characterization. The established microglial markers include transmembrane protein 119 (TMEM119) (55), P2RY12 (55), hexosaminidase subunit b (56,57) and ionized calcium-binding adapter molecule 1 (58). Microglia undergo adaptive morphological changes under varying conditions; healthy retinal microglia display a ramified morphology, whereas activated microglia in retinal injury adopt an amoeboid shape (59).

## 3. Mechanisms of microglial involvement in DR

### Impact of a hyperglycaemic microenvironment on microglial activation

**Figure 3:**
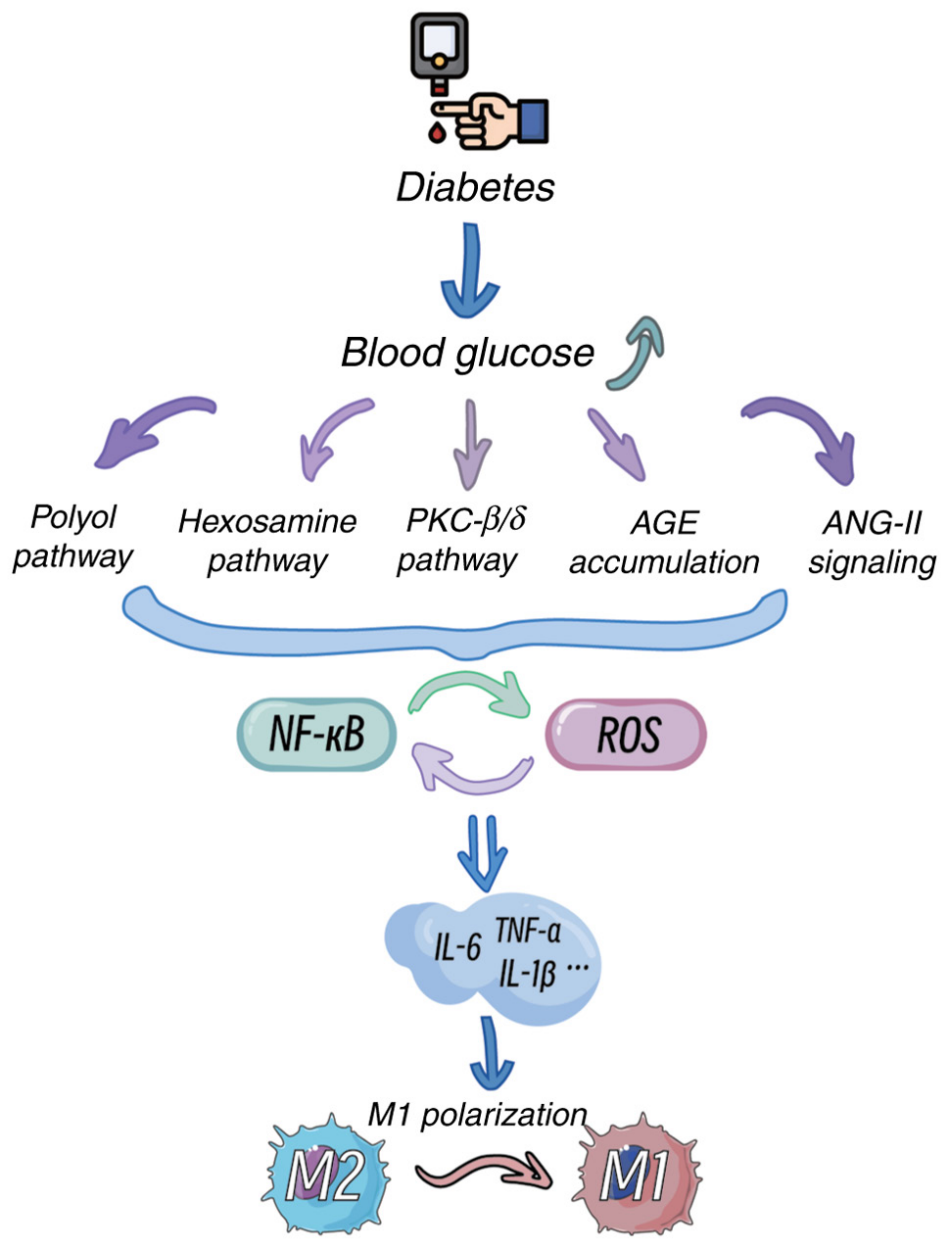
Effects of hyperglycaemic microenvironment on the activation of microglia. Diabetes leads to elevated blood glucose levels, which activate several metabolic pathways: the polyol pathway, hexosamine pathway, PKC-β/δ pathway, AGE accumulation and ANG-II signalling. These pathways converge to activate NF-κB and increase ROS production. The interplay between NF-κB and ROS creates a positive feedback loop, further amplifying the inflammatory response. This results in the release of pro-inflammatory cytokines such as IL-6, TNF-α and IL-1β, ultimately promoting M1 polarization of immune cells and impairing M2 polarization. AGE, advanced glycation end products; ANG-II, angiotensin II; ROS, reactive oxygen species.

The pathogenesis of DR is closely associated with chronic hyperglycaemia (2). Hyperglycaemia induces oxidative stress and inflammatory responses through multiple pathways, including the polyol pathway, hexosamine pathway, protein kinase C (PKC) activation, angiotensin II (ANG-II) signalling and advanced glycation end product (AGE) accumulation, thereby driving microglial activation (Fig. 3) (60-63). Among these pathways, the polyol pathway increases the intracellular osmotic pressure, leading to cellular oedema and rupture, whereas activated microglia participate in inflammatory processes by inhibiting the tricarboxylic acid cycle (61). Activation of the hexosamine pathway significantly elevates reactive oxygen species (ROS) levels, enhances the proinflammatory phenotype of microglia and exacerbates damage to the retinal NVU (63). Additionally, hyperglycaemia activates the PKC-β/δ pathway, triggering p38/MAPK-mediated oxidative stress and caspase-dependent apoptotic signalling, resulting in pericyte loss and microaneurysm formation (60,64,65). ANG-II and AGEs further amplify inflammatory cascades by activating the NF-κB pathway and binding to the receptor for AGEs (RAGE), respectively, promoting VEGF release and increased vascular permeability (64,66-69).

Clinical studies have indicated that under hyperglycaemic conditions, microglia rapidly activate and release various inflammatory cytokines, such as TNF-α, IL-1β and IL-6, forming a proinflammatory cycle (70-72). In diabetic patients with poor glycaemic control, retinal microglia exhibit significantly elevated activation levels, predominantly displaying the M1 proinflammatory phenotype during early DR stages, which exacerbates local inflammation. As the disease progresses, the capacity for M2 reparative phenotypic transformation diminishes, leading to impaired retinal repair functions (73,74).

### Retinal microglial effects on retinal vasculature

Hyperglycaemia-induced microglial activation serves as a central link connecting metabolic dysregulation and retinal vascular injury. Activated M1-like microglia disrupt BRB function and promote pathological neovascularization through the release of inflammatory mediators and the modulation of key signalling pathways (75,76). This process involves the destabilization of NVU homeostasis, endothelial cell dysfunction and immune microenvironment dysregulation, ultimately leading to the characteristic vascular pathologies of DR (77) Thus, microglia serve dual roles in both maintaining retinal homeostasis and driving BRB breakdown.

The dual regulatory role of retinal microglia in BRB homeostasis is central to vascular pathology in DR. While maintaining ocular immune privilege through selective molecular transport regulation (34,35), activated microglia exhibit paradoxical effects. Under hyperglycaemic conditions, M1-like microglia disrupt BRB integrity via Toll-like receptor 4 (TLR4)/myeloid differentiation primary response 88 (MyD88)/NF-κB signalling, promoting IL-1β secretion and subsequent downregulation of endothelial tight junction proteins (ZO-1 and claudin-5) (78-80). By contrast, quiescent microglia enhance BRB stability by upregulating these junctional complexes (80). This functional dichotomy extends to cellular interactions; microglial phagocytosis of endothelial cells and complement-mediated activation of RPE cells exacerbate vascular leakage, while their debris clearance and NVU maintenance functions provide protective effects (41,81-86). Pharmacological interventions, such as asiatic acid, demonstrate therapeutic potential by modulating the balance of microglial polarization in DR (79).

These microglial-mediated vascular barrier alterations create a proangiogenic microenvironment that synergistically promotes the pathological neovascularization processes. In retinal diseases, microglial activation promotes inflammatory factor production and BRB damage, leading to a hypoxic retinal microenvironment. This hypoxia further drives aberrant neovascularization, exacerbating disease progression (87).

Microglia exert both direct and indirect effects on pathological neovascularization in DR. A previous study demonstrated that microglia cultured under hypoxic conditions *in vitro* exhibit significantly increased expression of proangiogenic factors, particularly VEGF and insulin-like growth factor 1 (IGF-1) (88). This finding highlights that hypoxia induces microglia to upregulate these key factors, thereby promoting retinal neovascularization (90). These observations suggest that in PDR, microglia not only mediate retinal neovascularization but also amplify this process by exacerbating hypoxia (89,90).

Microglia regulate pathological neovascularization through the release of inflammatory mediators. VEGFR1 expression increases with M1-like microglial activation, promoting pathological angiogenesis (89). Under pathological conditions, VEGFR1 activation further upregulates VEGF-A, TNF-α and placental growth factor (PGF), forming a positive feedback loop that perpetuates neovascularization (91). As microglia accumulate around nascent vessels, they release additional angiogenic factors through distinct subtypes. Activated microglia engaging in receptor-interacting protein 1 and 3 (RIP-1 and -3) signalling undergo necroptosis, releasing fibroblast growth factor 2 to stimulate retinal neovascularization (92). Colony-stimulating factor 1 receptor (CSF1R)-positive microglia secrete TNF-α to promote angiogenesis (93). Basigin 2-enriched microglia clustered around sprouting vessels increase IGF-1 secretion, driving neovascular growth (94). Galectin 3 binding protein-overexpressing microglia increase the levels of hypoxia-inducible factor 1α (HIF-1α), VEGF-A and matrix metalloproteinases (MMP-2/MMP-9), facilitating pathological angiogenesis (95).

Certain microglial subtypes mitigate neovascularization. Fas ligand-positive (FasL+) microglia induce the apoptosis of Fas+ endothelial cells and phagocytose them (96), whereas thrombospondin-1-positive (Trp-1+) microglia suppress endothelial proliferation and migration via microRNA-enriched exosomes and SMAD3 signalling in endothelial cells (97).

Microglia also regulate retinal neovascularization through specific signalling pathways. Enhanced CD200R-CD200 (on microglia and endothelial cells, respectively) interactions critically drive angiogenesis (98). Neuropilin-1 (NRP-1) expression in microglia indirectly supports neovascularization, as NRP-1 deficiency reduces perivascular microglial recruitment and angiogenesis (99,100). Secreted phosphoprotein 1 mediates microglia-endothelial crosstalk to promote endothelial proliferation (101). Galectin-3 binds jagged-1 to inhibit Notch signalling, thereby enhancing endothelial cell proliferation (102).

These findings underscore the dual roles of microglia in retinal vascular pathology. The phenotypic heterogeneity of microglia and their interactions with hypoxic microenvironments exacerbate vascular lesions, suggesting that targeting microglial polarization or key signalling pathways may offer novel therapeutic strategies for DR.

### Dual roles of microglia in neuroprotection and neurotoxicity in DR

As resident immune cells of the retina, microglia serve critical roles in maintaining retinal homeostasis and responding to injury. Under physiological conditions, microglia exert neuroprotective effects by releasing anti-inflammatory factors, such as IL-10 and TGF-β, to suppress inflammatory responses and safeguard retinal neurons (103). In early DR stages, microglia further protect retinal ganglion cells from hyperglycaemia-induced damage by activating the PI3K/Akt signalling pathway to secrete neurotrophic factors, such as brain-derived neurotrophic factor, thereby promoting the repair and regeneration of injured neurons (104).

During DR progression, however, microglia shift towards a proinflammatory phenotype, contributing to neural injury. Under hyperglycaemic conditions, activated microglia release excessive proinflammatory cytokines, including TNF-α and IL-1β, which disrupt synaptic connectivity and impair neuronal signalling, exacerbating retinal inflammation and neurodegeneration (16). The number of activated microglia increases significantly in DR retinas, where they sustain inflammatory factor secretion, which not only directly damages neurons but also compromises BRB integrity, facilitating peripheral immune cell infiltration. These processes establish a self-perpetuating 'inflammation-injury' cycle, ultimately accelerating degenerative changes in retinal neural tissue (105).

In the systemic hyperglycaemic milieu, retinal microglia generate excessive ROS, which directly damage neuronal cell membranes and mitochondria (106). Additionally, hyperglycaemia-activated microglia release nitric oxide, which induces neuronal apoptosis by mediating glutamate excitotoxicity and promoting the nuclear accumulation of GAPDH (74).

In summary, microglia exhibit dual mechanisms in DR, balancing neuroprotection and neurotoxicity. A deeper understanding of these mechanisms may inform novel therapeutic strategies to delay DR progression.

### Altered microglial intercellular interactions in DR

During retinal vascular development, macroglia, such as astrocytes, form scaffold-like structures with loose ensheathment around nascent vessels, whereas Müller cells collaborate with astrocytes to maintain BRB integrity (107-109). Microglia further support vascular growth and stabilization through dynamic interactions with these macroglial cells (110).

### Microglial-Müller cell interactions

Müller cells respond to microglial activation at the molecular and functional levels, enhancing microglial activation and migration through bidirectional signalling between the two cell types (111). Additionally, Müller cells amplify inflammatory responses across retinal layers via chemotactic and adhesive cell-cell contacts, mobilizing further microglial migration (111). Activated microglia release inflammatory factors such as TNF-α, which in turn activate Müller cells, triggering their proliferation and additional cytokine release. This mutual activation forms a vicious cycle that exacerbates retinal inflammation. Activated Müller cells further release ATP, promoting microglial activation via the P2X7 receptor pathway (112). These reciprocal interactions not only disrupt the BRB, increasing vascular permeability and retinal oedema, but also impair neural signalling and worsen neurodegeneration (113). Moreover, inflammatory factors (e.g., TNF-α and IL-1β) released through microglial-Müller cell crosstalk directly damage retinal neurons, accelerating DR progression (114).

### Microglia-astrocyte interactions

In the early DR stages, activated retinal microglia release inflammatory mediators that stimulate astrocyte activation, driving their proliferation and further cytokine secretion (115). Hypoxia-stressed astrocytes in DR also rapidly activate and secrete VEGF and inflammatory factors to promote pathological neovascularization (115). Concurrently, microglial activation synergistically enhances angiogenesis, hastening DR progression. Mutual activation of microglia and astrocytes contributes to BRB disruption (116). Microglia indirectly regulate vascular development by modulating the spatial patterning of astrocytes (117). Activated astrocytes adjacent to vascular endothelial cells release vasoactive substances, such as VEGF, increasing vascular permeability and exacerbating retinal oedema (115).

## 4. Signalling pathways of microglia in DR

### TLR4/MyD88/NF-κB p65 signalling pathway

**Figure 4:**
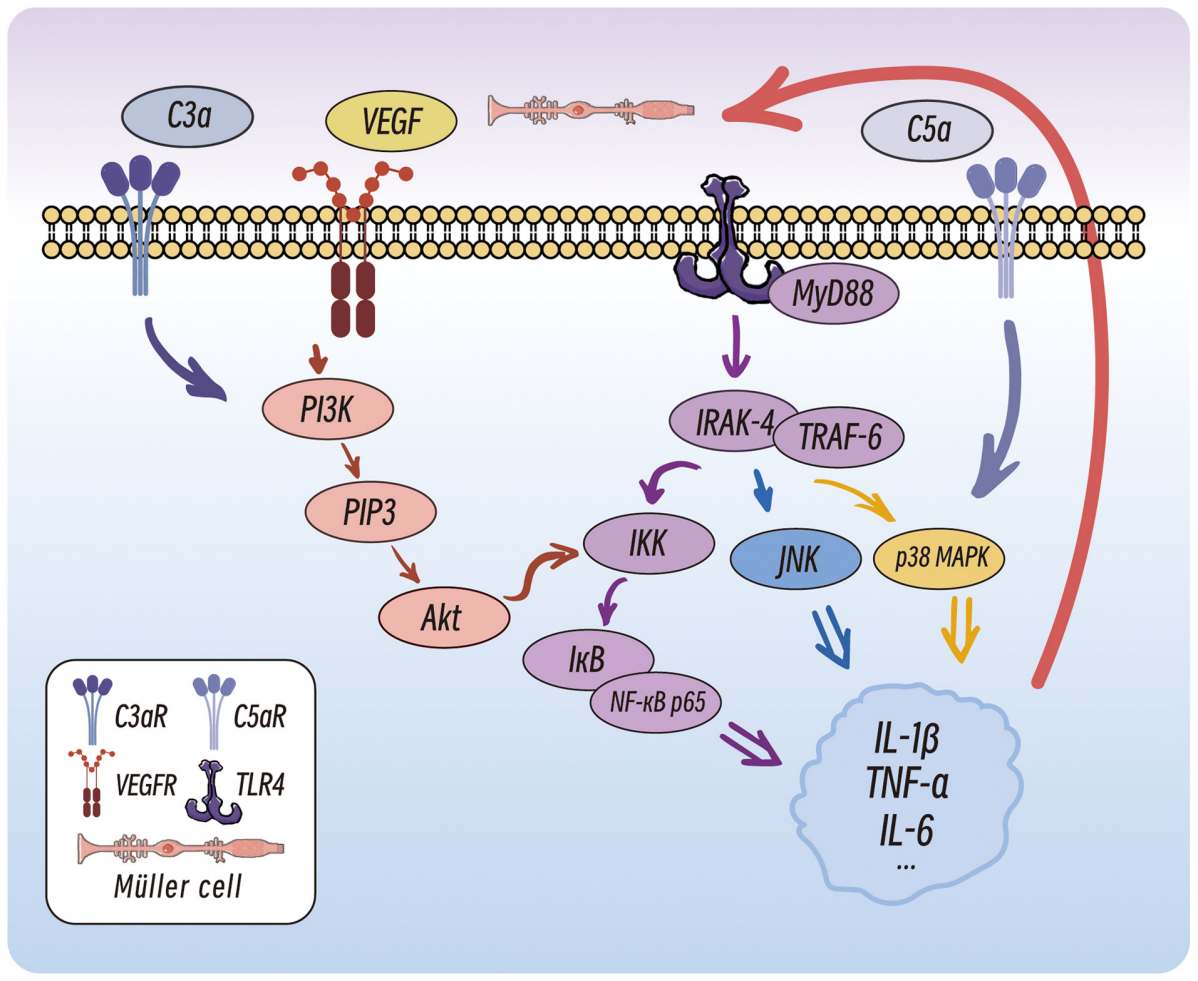
Signalling pathways and cellular interactions mediated by microglia in DR. In DR, microglia express receptors such as C3a and C5a receptors, VEGFR and TLR4. The activation of these receptors triggers intracellular signalling pathways which subsequently activate downstream kinases. These signalling cascades lead to the activation of transcription factors such as NF-κB, resulting in the production and release of pro-inflammatory cytokines including IL-1β, TNF-α and IL-6. The released pro-inflammatory cytokines then act on Müller cells, promoting the release of VEGF. This process highlights the role of microglia in mediating inflammatory responses and angiogenesis in DR through these signalling pathways. DR, diabetic retinopathy; TLR-4, Toll-like receptor 4; MyD88, myeloid differentiation primary response 88; IRAK-4, interleukin-1 receptor-associated kinase 4; TRAF6, TNF receptor-associated factor 6; IKK, IκB kinase.

The activation of retinal microglia involves multiple signalling pathways, with the TLR4/MyD88/NF-κB p65 pathway serving a pivotal role (Fig. 4) (118). M1-polarized microglia recognize pathogen-associated molecular patterns (PAMPs) and damage-associated molecular patterns (DAMPs) through TLRs, particularly TLR4. MyD88 binds to TLR4 via its Toll/interleukin-1 receptor domain to initiate downstream signalling, which recruits interleukin-1 receptor-associated kinases (IRAK), such as IRAK-4. This leads to IRAK phosphorylation and activation, followed by interaction with TNF receptor-associated factor 6 (TRAF6), which activates the IκB kinase (IKK) complex. IKKβ phosphorylates IκBα, a cytoplasmic inhibitor of NF-κB, resulting in the release and nuclear translocation of NF-κB. Nuclear NF-κB binds to promoters of proinflammatory genes (including IL-1β, IL-6 and TNF-α), driving cytokine production (119). These cytokines further activate the TLR4/MyD88/NF-κB p65 pathway, creating a self-amplifying inflammatory loop (120).

### MAPK signalling pathway

The MAPK pathway also plays a critical role in microglial activation. In DR, hyperglycemia-induced oxidative stress and AGEs first trigger Toll-like/IL-1 receptors on retinal microglia. Receptor engagement leads to phosphorylation of IRAK-1/4, which forms a complex with TRAF6 and initiates downstream MAPK cascades, sequentially activating the MKK3/6-p38 MAPK and MKK4/7-JNK pathways (121). These cascades drive sustained synthesis of pro-inflammatory and pro-angiogenic factors (TNF-α, IL-1β, VEGF), disrupt the blood-retinal barrier, and accelerate neovascularization, positioning them at the core of DR pathogenesis (83,122).

### Interactions with Müller cells and endothelial cells

Proinflammatory IL-1β binds to IL-1 receptors (IL-1Rs) on Müller cells, enhancing VEGF transcription and secretion (78). VEGF binds to VEGFR2 on endothelial cells, recruiting PI3K to generate phosphatidylinositol (3,4,5)-trisphosphate (PIP3) and activate Akt (123). Akt phosphorylates the IKK complex, accelerating IκB degradation and NF-κB nuclear translocation (123). Additionally, VEGF disrupts the BRB by activating the MAPK1 and p38 pathways, which downregulates tight junction proteins (ZO-1 and claudin-5) in endothelial cells (124,125).

### Complement system activation

The C3a and C5a complement components activate microglia by binding their respective receptors, inducing the secretion of inflammatory mediators (126). As a key component of innate immunity, the complement system regulates tissue homeostasis and angiogenesis (127). The activation of complement receptors on microglia further stimulates the NF-κB and MAPK pathways, forming a positive feedback loop that exacerbates microglial activation and inflammation (128). The terminal stage of complement activation generates the membrane attack complex (MAC), which inserts into cell membranes, causing pore formation and cell death. Increased MAC levels are observed in the retinas of patients with DR and a STZ-induced diabetic rat model, directly damaging neuronal membranes and impairing function (129).

### Hyperglycaemia-specific signalling features

Under hyperglycaemic conditions, microglia upregulate TLR4 expression, which increases their sensitivity to PAMPs and DAMPs, thereby sustaining chronic inflammation (119). Concurrently, high glucose activates oxidative stress pathways (such as NADPH oxidase) in microglia, leading to excessive ROS production that amplifies inflammatory responses (130,131).

Studies have demonstrated that inhibition of the NF-κB pathway significantly reduces microglial activation and inflammation in diabetic retinas (83,132-134). Thus, targeting NF-κB and MAPK signalling or blocking complement activation may represent novel therapeutic strategies for DR.

## 5. Potential therapeutic applications of microglia in DR

Current standard treatments for PDR include panretinal photocoagulation (PRP), intravitreal anti-VEGF agents (used alone or combined with surgery), angiopoietin inhibitors and vitrectomy (135,136). While these approaches provide clinical benefits, they fail to address the underlying disease mechanisms (137,138). Furthermore, therapeutic efficacy varies among patients, with suboptimal responses observed in certain cases. PRP, while effective in eliminating neovascularization, may stimulate neuroglial hyperplasia, which damages surrounding healthy retinal tissue and causing peripheral vision loss or permanent retinal scarring. PRP should be administered with caution in patients who still rely on peripheral vision or in those with extensive retinal scarring or severely impaired retinal function (139). Anti-VEGF therapy, despite its effectiveness, carries risks of exacerbating inflammation and accelerating cataract formation (140). For patients with tractional retinal changes or vitreoretinal traction, anti-VEGF therapy may exacerbate the traction and increase the risk of tractional retinal detachment (135). These limitations underscore the need for improved treatment strategies (141,142).

Abnormal aggregation and polarization of microglia serve pivotal roles in DR pathogenesis (143). Targeting microglial activation may thus serve as a promising adjunctive therapy for PDR, offering a novel approach that is complementary to existing treatments.

### Reducing activated microglial populations

Previous studies have demonstrated that certain anti-VEGF therapies influence microglial activity (89,144,145). For example, Arias *et al* (145) reported that the anti VEGF agent aflibercept modulates microglial activity and increases the proportion of quiescent microglia, thereby promoting the regression of retinal neovascularization in oxygen-induced retinopathy models. Ranibizumab is a recombinant humanized monoclonal antibody fragment (Fab fragment) that binds with high affinity to and inhibits all biologically active forms of VEGF-A, and has also been shown to inhibit the expansion of activated microglia (146).

In addition to anti-VEGF therapies, various novel approaches have demonstrated the potential to suppress or reduce activated microglia, thereby attenuating pathological retinal angiogenesis. These approaches include melatonin (147), TGF-β-activated kinase 1 inhibitors (148), diphtheria toxin (149), CSF1R antagonists (149), microglial replacement strategies (clearing diseased microglia and repopulating with healthy donor-derived cells) (150), cyanidin-3-O-glucoside (151), honokiol (152), KC7F2 antagonists and HIF1α antagonists (153). These therapeutic interventions target the activation state of microglia, offering diverse strategies and options for DR treatment.

### Promoting M1-to-M2 phenotype shift

M2-polarized microglia exhibit inhibitory effects on DR progression, making the conversion of M1 microglia to M2 microglia a potential therapeutic strategy to suppress DR pathogenesis (154). Sun *et al* (155) suggested that ferulic acid induces this phenotypic shift, although its clinical application in patients with DR remains unexplored. However, excessive M2 polarization may paradoxically drive a proangiogenic shift, promoting retinal neovascularization (156,157). Thus, therapeutic strategies should aim to balance M1/M2 polarization by maintaining a specific ratio, although the optimal range requires further investigation.

Arginase-1, an enzyme involved in the urea cycle and immune regulation, serves a key role in regulating microglial polarization (158-160). While preliminary evidence indicates the influence of arginase-1 on microglial phenotypes, the precise molecular mechanisms and functional outcomes need further exploration (43). Additionally, melatonin has been shown to promote M1-to-M2 conversion via regulatory T-cell-mediated pathways, facilitating tissue repair (35).

### Inhibiting proangiogenic cytokine secretion

In addition to TNF-α and IL-1β, VEGF is a key proangiogenic biomarker secreted by microglia that promotes retinal neovascularization through direct or indirect pathways (87,89). Anti-VEGF therapies, including monoclonal antibodies (e.g., bevacizumab and ranibizumab), fusion proteins (e.g., aflibercept), tyrosine kinase inhibitors (e.g., sunitinib, sorafenib and pazopanib), receptor-targeting antibodies (e.g., ramucirumab) and aptamers (e.g., pegaptanib), block angiogenesis-related cytokines via diverse mechanisms to inhibit endothelial cell proliferation and pathological neovascularization (161). Additionally, several pharmacological and biological agents, such as intravitreal triamcinolone acetonide-conjugated dendritic nanoparticles (162), the FAD286 aldosterone synthase inhibitor (163), ω-3 polyunsaturated fatty acids (ω-3 PUFAs) (93,164), the NLY01 glucagon-like peptide-1 receptor agonist (96), chlorogenic acid (165), erianin (166) and celastrol (167), have demonstrated efficacy in suppressing DR progression to PDR by inhibiting microglial secretion of proangiogenic factors or modulating their interactions with the retinal vasculature.

### Risks and challenges in microglial-targeted therapies

Although microglial-targeted therapies show promising potential for DR treatment, significant risks and challenges are faced. Under physiological conditions, microglia perform critical functions, such as immune surveillance and maintenance of retinal microenvironment homeostasis (168). However, pathological overactivation or excessive suppression of microglia may lead to adverse outcomes (169).

The activation states and functional regulation of microglia are highly complex. Single intervention approaches often fail to precisely steer the polarization of microglia towards a therapeutically beneficial phenotype, limiting treatment efficacy (170). In addition, excessive suppression of microglial activation and potential reduction of inflammation may compromise the normal immune surveillance capacity of microglia, increasing susceptibility to infections (171). Excessively suppressing microglial activation may also impair the role of microglia in retinal microenvironment maintenance and repair, potentially triggering secondary complications (52).

Furthermore, the long-term efficacy and safety of microglial-targeted therapies require further validation. Current studies predominantly focus on short-term outcomes, with limited experimental or clinical data addressing critical issues such as long-term functional changes in microglia, delayed adverse effects or persistent impacts on retinal physiology. This includes research demonstrating the significant therapeutic potential of the NOX1/4 inhibitor GKT137831 (Setanaxib) in diabetic retinopathy models, which effectively reduces Iba1-positive microglia population, lowers their production of reactive oxygen species, and decreases expression of inflammatory factors such as VEGF, IL-6 and TNF-α (74,170,172,173,174).

Advancements in localized drug delivery technologies, however, offer new avenues to address these challenges. For example, nanoparticle-based retinal targeting systems are rapidly evolving. By engineering nanocarriers for precise intraretinal drug delivery and sustained release, these systems increase the drug concentration at lesion sites while minimizing systemic side effects (175). Such innovations may mitigate the risks associated with microglial modulation, improving both therapeutic efficacy and safety, thereby strengthening the foundation for the clinical translation of microglial-targeted strategies in DR (176).

## 6. Conclusions and perspectives

In DR, microglia serve dual roles; microglia migrate and proliferate to participate in ocular immune privilege, vascular debris clearance and retinal protection/repair, and simultaneously contribute to BRB disruption via activation of the TLR4/MyD88/NF-κB p65 signalling pathway. Furthermore, activated microglia promote NVU dysfunction and pathological neovascularization through the release of inflammatory mediators and the modulation of signalling pathways.

Given this dual functionality, microglial-targeted therapies, such as reducing activated microglial populations, promoting M1-M2 phenotypic shifts and inhibiting proangiogenic cytokine secretion, offer a novel approach to DR management. These strategies may complement existing treatments, such as PRP and intravitreal anti-VEGF injections, while mitigating their side effects. However, as microglia are also present in the CNS, therapeutic interventions must be localized to the retina to avoid systemic impacts. The development of precise microglial-targeted therapies remains challenging owing to an incomplete understanding of the exact contributions of microglia to DR progression.

Future research should prioritize comprehensive investigations into microglial subtypes, their functional dynamics in the diabetic retina and their interactions with other glial cells. This includes developing strategies to precisely regulate microglial activity without off-target effects and exploring microglia-derived biomarkers for early DR detection and intervention. Advances in these areas will provide transformative therapeutic solutions for this high-incidence disease that severely impacts quality of life.

## Data Availability

Not applicable.
